# Dimensionality reduction in hyperspectral imaging using standard deviation-based band selection for efficient classification

**DOI:** 10.1038/s41598-025-21738-4

**Published:** 2025-10-03

**Authors:** Wolfgang Kurz, Kun Wang, Furkan Bektas, Changyan Zhu, Emre Kariper, Xingchen Dong, Michael Kurz, Martin Jakobi, Danny Baranes, Alexander W. Koch

**Affiliations:** 1 Department of Electrical Engineering, Institute for Measurement Systems and Sensor Technology, Technical University of Munich, Munich, 80333 Germany; 2 School of Physical & Mathematical Sciences, Nanyang Technological University, Nanyang, 637371 Singapore; 3 Department of Mechanical, Automotive and Aeronautical Engineering, Hochschule München University of Applied Sciences, Munich, 80335 Germany; 4 Department of Molecular Biology, Ariel University, Ari’el, 40700 Israel

**Keywords:** Imaging, Microscopy

## Abstract

Hyperspectral imaging generates vast amounts of data containing spatial and spectral information. Dimensionality reduction methods can reduce data size while preserving essential spectral features and are grouped into feature extraction or band selection methods. This study demonstrates the efficiency of the standard deviation as a band selection approach combined with a straightforward convolutional neural network for classifying organ tissues with high spectral similarity. To evaluate the classification performance, the method was applied to eleven groups of different organ samples, consisting of 100 datasets per group. Using the standard deviation is an effective method for dimensionality reduction while maintaining the characteristic spectral features and effectively decreasing data size by up to 97.3%, achieving a classification accuracy of 97.21% compared to 99.30% without any processing. Even in comparison with mutual information– and Shannon entropy–based band selection methods, the standard deviation exhibited superior stability and efficiency while maintaining equally high classification accuracy. The results highlight the potential of dimensionality reduction for hyperspectral imaging classification tasks that require large datasets and fast processing speed without sacrificing accuracy.

## Introduction

**Fig. 1:**
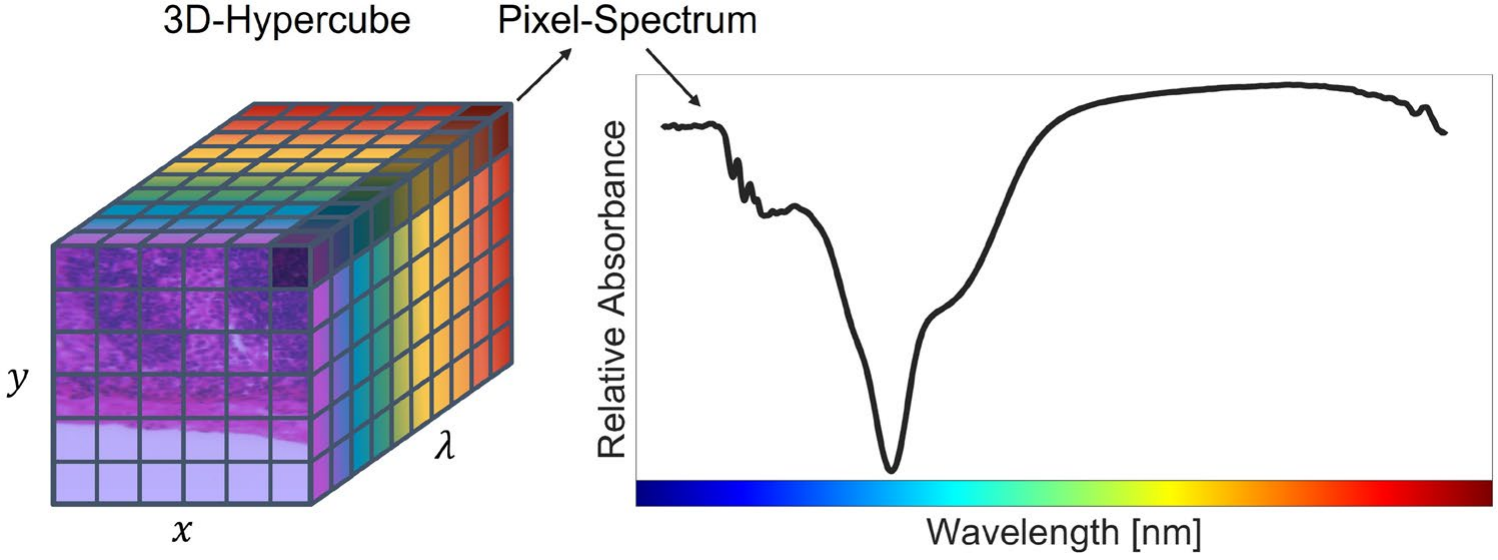
Hypercube representation of a lymph node section with spatial dimensions (y, x) and spectral dimension $(\lambda )$. The hypercube consists of a full spectrum for each spatial pixel. The spectrum of the top-right pixel is shown on the right, illustrating its relative absorbance across the measured wavelength range. The data were acquired using the hyperspectral imaging (HSI) setup described in this study.

Hyperspectral imaging (HSI) is a non-invasive, contact- and label-free technique that gathers spatial and spectral information from a sample^1^. Hence, light interacting with the sample is transmitted or reflected to the hyperspectral imager, which provides spectral data for each pixel in an image. The spectral data typically covers a range from the ultraviolet (UV) to the near-infrared (NIR) range^2^. The combination of two-dimensional spatial and one-dimensional spectral data forms a three-dimensional hypercube, as shown in Fig. 1.

Each object in a scanned scene has a unique spectral signature, providing detailed information about its composition that enables accurate identification of on-site materials. This unique property makes HSI a powerful technology with a wide range of applications in remote sensing^2,3^, such as agriculture monitoring^4,5^, environmental sensing^6,7^, geological mapping^8,9^, as well as in food quality assessment^6,10^, industrial manufacturing^11,12^, forensic science^13,14^, and biomedical applications^15,16^.

Fast and precise analysis is particularly important in the medical field, where a rapid diagnosis can significantly impact patient outcomes. HSI represents an optimal method for the reliable classification of biomedical samples, avoiding histopathology or invasive diagnostic procedures, leading to early disease detection, more effective treatments, and improved patient care^17^. For instance, utilizing HSI alongside deep learning methods shows an enhanced classification of skin cancer features compared to traditional RGB imaging by extracting detailed spectral bands, reducing image noise, and highlighting lesion features^18^. Intraoperative imaging using HSI assists surgeons in real-time differentiating between healthy and diseased tissue while remaining label-free^19,20^. In ophthalmology, HSI can identify retinal diseases like age-related macular degeneration by studying autofluorescence of the ocular fundus, providing important insights into the pathogenesis^21^. HSI provides precise information on various perfusion and substance parameters, such as tissue oxygen saturation, tissue hemoglobin index, or tissue water index, revealing valuable information about wound healing processes^22,23^. Additionally, studying single cells with HSI leads to a better understanding of the underlying processes of disease progression, the cellular response to drug therapy, and the fundamental biology of various cell types^24^. With the increasing number of applications and the complexity of HSI, there is a growing need for efficient high-dimensional data processing to decrease computational load and analyze the results in real-time. Additionally, many spectral bands are highly correlated, resulting in information overlap^25^. Dimensionality reduction is a common approach to address these issues by extracting or preserving a dataset’s significant and unique spatial-spectral features while eliminating irrelevant and redundant spectral bands^26^. This challenge is especially relevant today, as deep learning methods have become increasingly popular for HSI classification in all areas, with numerous architectures introduced in recent years^27–29^. In particular, modern convolutional neural networks, recurrent models, and transformer-based networks achieve unmatched accuracy in HSI classification tasks^30–32^, but are highly complex. Choosing the appropriate dimensionality reduction method is still a major challenge in HSI since there is no standard procedure for selecting the optimal method. This decision depends on various influencing factors, such as the type of dimensionality reduction, area of application, environmental circumstances, technical specifications, and the level of reduction without affecting the classification results^33^.

Several dimensionality reduction methods have been extensively applied, especially in time-critical or resource-limited scenarios. In the field of Earth observation, where HSI originally emerged, reducing the high spectral dimensionality is essential for tasks such as real-time target detection, onboard processing, and large-scale land classification^34^. Among the most commonly used dimensionality reduction methods are Principal Component Analysis (PCA), Independent Component Analysis (ICA), and Linear Discriminant Analysis (LDA)^35^.

Lupu et al.^36^ performed a comprehensive study comparing eight unsupervised dimensionality reduction methods, including PCA, ICA, Orthogonal Subspace Projection (OSP), Locality Preserving Projections (LPP), Very Sparse Random Projection (VSRP), Non-negative Matrix Factorization (NMF), Deep Belief Networks (DBN), and Convolutional Autoencoders (CAE) using HSI from earth observation satellites. Their performance metrics included computation time, reconstruction accuracy, independence of components, sensitivity to artifacts, target detection performance, and classification performance. They concluded that none of these methods was superior to the other. Instead, the effectiveness of each technique depends on the area of application, highlighting the challenge of finding the optimal dimensionality reduction method that balances all requirements.

A more advanced dimensionality reduction strategy incorporates information-theoretic principles to identify the most class-relevant spectral features. Islam et al.^37^ proposed a hybrid method combining a noise-adjusted transform—Minimum Noise Fraction (MNF) with mutual information (MI) ranking and the Minimum Redundancy Maximum Relevance (mRMR) criterion. Their approach selected the most informative and least redundant spectral components using publicly available data. A subsequent Support Vector Machine (SVM) is used for classification. The results outperformed PCA, LDA, and other conventional methods for all tested datasets, reaching classification accuracies of 97.44% for dataset 1, 99.71% for dataset 2, and 98.35% for dataset 3. While MI-based selection offers high classification results, it also comes with high complexity, relying on labeled data, requiring estimating probability distributions in high-dimensional space, and several processing steps involving transformations, entropy calculations, and optimization.

In biomedical hyperspectral imaging, clustering-based band selection has proven to be an effective unsupervised approach. Zhang et al.^38^ introduced a novel algorithm that groups highly correlated or redundant spectral bands based on similarity metrics and selects representative bands from each cluster. This Data Gravitation and Weak Correlation Ranking (DGWCR) approach was applied to in vivo tissue scans and medical HSI datasets. It preserved diagnostically relevant spectral content while significantly reducing data dimensionality. Unlike feature extraction techniques, clustering-based methods maintain the original spectral bands, improving interpretation for clinical or biological insights. Experimental results demonstrated that the reduced set of selected bands can surpass the full-spectrum data in classification accuracy for tissue differentiation tasks. This improvement results from removing noise and spectral redundancy, which often degrade the performance of downstream classifiers. However, clustering-based approaches like DGWCR rely on the assumption that meaningful spectral features form clearly defined clusters, which is not always true in complex biomedical data. Additionally, selecting the number of clusters and tuning the similarity metrics are typically set individually and are also sensitive to spectral noise and sample variability, which can lead to inconsistent band selection.

Wang et al.^39^ developed a Deep Margin Cosine Autoencoder (DMCA) for tumor tissue classification in medical HSI. The method integrates a deep autoencoder for spectral compression with a cosine-margin loss function to enhance class separability in the latent space. The encoder learns a compact representation of the spectral data, while the decoder reconstructs the input, retaining meaningful features. Simultaneously, a supervised classification optimizes the embedding to distinguish between tumor and healthy tissue regions. The DMCA model was evaluated on hyperspectral biopsy images and demonstrated superior performance compared to several conventional methods, including the SVM, Convolutional Neural Network (CNN), and Fusion Spectral–Spatial Transformer (FUST), among others. One of the two datasets achieved classification accuracies of 98.41% for normal tissue, 99.97% for tumor tissue, 99.93% for hypervascularized tissue, and 99.81% for labeled background regions. This highlights the potential of non-linear, task-driven dimensionality reduction in capturing subtle spectral differences in complex biomedical data. Despite their effectiveness, deep autoencoders require extensive labeled datasets, architecture tuning, and substantial computational resources, factors that may hinder deployment in clinical environments. Furthermore, the resulting features are abstract and lack direct spectral interpretability, which complicates their integration into diagnostic pipelines that require transparency.

While advanced methods such as deep autoencoders or MI–based selection yield high classification performance, they often require high computational cost, large labeled datasets, or sacrifice interpretability due to spectral mixing or complex feature transformations. In contrast, methods like clustering-based band selection or PCA offer better interpretability but assume linear relationships and may not capture non-linear patterns while prioritizing high-variance variables, potentially ignoring meaningful low-variance variables. These challenges highlight the need for a straightforward, rapid, and easily implementable alternative that preserves the physical identity of spectral bands while reducing redundancy.

The present study applies the standard deviation (STD) for band selection in conjunction with a straightforward neural network to demonstrate the effectiveness of specific band selection, even in cases where organ tissue exhibits strong visual and spectral similarity. Our results imply that only a small subset of spectral bands with maximum information content is sufficient for accurate classification.

To further demonstrate the effectiveness of the STD-based method, three common band selection methods are implemented for comparison, including random band selection (RBS), MI-based selection, and Shannon entropy-based selection. The findings demonstrate the superiority of the STD-based method and suggest that significant data reduction is feasible without compromising classification quality, enabling more efficient and scalable HSI applications. This work evaluates a simple and interpretable statistical criterion for band selection in complex biomedical hyperspectral imaging scenarios, offering an efficient alternative to more computationally intensive methods.

## Materials and methods

### Hyperspectral imaging setup

**Fig. 2:**
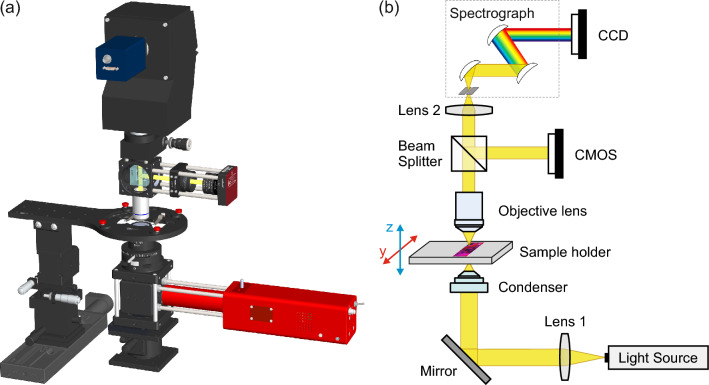
(**a**) 3D model of the HSI setup. (**b**) Schematic of the HSI setup and its optical path consisting of two collimating lenses (Lens 1, Lens 2), a condenser, an objective lens with 100$\times$ magnification, a beam splitter, a CMOS camera, and a spectrograph with a CCD camera. The sample is placed on a sample holder.

An HSI system is used to collect the hypercubes of each sample. It comprises an HSI microscope, a broadband light source, and a sample holder, as shown in Fig. 2.

The broadband light source (SLS201L/M, Thorlabs) features a stabilized tungsten-halogen bulb with a blackbody radiation spectrum ranging from 360 nm to 2600 nm and integrated collimating optics (Lens 1, SLS201C, Thorlabs). The light beam is guided into an achromatic condenser (CSC2001, Thorlabs) using a silver mirror (PFR10-P01, Thorlabs). The condenser gathers the collimated light and focuses it onto the sample. A high-precision motorized sample holder (PI miCos GmbH, VT-80, DC) moves the sample along the y direction to allow line scanning. The step size is 0.5 $\mu$m per scanned line. The samples are magnified using a 100$\times$ N PLAN objective lens (N PLAN EPI 100$\times$/0.85, Leica) with a numerical aperture (NA) of 0.85. The 100$\times$ magnification provides a high level of detail within the image, preserving spectral integrity and allowing for more comprehensive data collection. To ensure precise focusing and positioning before each scan, the light beam is additionally directed to a monochrome CMOS camera (CS165MU/M, Thorlabs) through a removable 50:50 beamsplitter (BS016, Thorlabs), generating a real-time image of the sample. The second lens (Lens 2, AC254-050-AB, Thorlabs) focuses the collimated light beam into the hyperspectral imager. The hyperspectral imager consists of a spectrograph (G4-330, Headwall Photonics) that disperses the light into its constituent spectral bands and focuses the dispersed light onto a CCD camera (RA1000m/D, Adimec), which covers the spectral range from 325 nm to 1056 nm with a resolution of 0.728 nm^40^. This configuration results in 1004 spectral channels with a total spectral range of 731 nm. Each scan produces a hypercube with dimensions of 64$\times$64$\times$1004, where the first two dimensions (64$\times$64) represent the spatial dimension (y scanning lines, x pixels), and the third dimension ($\times$1004) represents the spectral channels ($\lambda$). Each scan takes approximately 10 seconds, enabling comprehensive data acquisition while maintaining a manageable data size and efficient processing.

### Dimensionality reduction method

Dimensionality reduction is essential to facilitate the processing of complex high-dimensional data by eliminating redundancy in spectral data while preserving the spectral bands that contain the most information^26^. There are two categories of dimensionality reduction methods for HSI: feature extraction and band selection, also known as feature selection^41,42^. Feature extraction irreversibly transforms the original high-dimensional data into a lower-dimensional space by directly selecting a group of features based on specific criteria. Common feature extraction methods include PCA, ICA, LDA, MNF, and autoencoders^43–48^. However, these methods result in a loss of physical interpretability of the data as they alter the original spectral characteristics^49^. Band selection describes the process of selecting the most relevant spectral bands with the maximum information content for a specific task while discarding redundant bands to reduce dimensionality. This method effectively preserves the physical properties of the original hyperspectral data^33^. There are six types of band selection: ranking-based, searching-based, clustering-based, sparsity-based, embedding learning-based, and hybrid scheme-based methods^50^.

#### Standard deviation-based band selection

In this study, a ranking-based band selection using the STD is applied. Ranking-based methods evaluate each spectral band separately, generating a subset of bands that contain the most information^51^. The STD measures dispersion or variability in a set of data values ranging from 0 to $\infty$. A high STD indicates that the intensity values at different wavelengths are spread far from the mean, suggesting significant variability in the data^52^. This variability highlights a distinct spectral characteristic of the sample, demonstrating that utilizing the STD can offer a straightforward yet effective approach to dimensionality reduction. Importantly, this approach preserves the physical interpretability of the data without feature transformation that might obscure biological significance. Due to its simplicity, broad applicability, and computational efficiency, the STD is particularly well-suited as a band selection method. The presented method estimates the STD by sliding a window along the spectral axis, computing the STD for all pixels at each wavelength, and thereby identifying the spectral feature with the greatest variation. The size of the sliding window is set to 20 nm, 50 nm, and 100 nm. Window sizes smaller than 20 nm are avoided to prevent the selection of spectral areas with high noise caused by the HSI system instead of distinctive spectral features. The entire spectral range of 731 nm (325 nm to 1056 nm) is included as a reference for classifying organ samples. This method effectively reduces the dimensionality of hyperspectral data, enhancing the computational efficiency without compromising the accuracy and reliability of subsequent processing. The hyperspectral data cube is represented as $X \in \mathbb {R}^{y \times x \times \lambda }$, where *y* and *x* denote the spatial dimensions (height and width), and $\lambda$ is the total number of spectral bands. To identify the most informative spectral region, a sliding window of a fixed width *w* is applied along the spectral axis. For each window starting at index *s*, the corresponding sub-cube is defined as:

1 $$\begin{aligned} X_{s:e} = X[:, :, s:e], \quad \text {where } e = s + w. \end{aligned}$$

For each spectral band $\lambda$, the standard deviation $\sigma _\lambda$ is computed as:

2 $$\begin{aligned} \sigma _\lambda = \sqrt{\frac{1}{yx} \sum _{i=1}^{y} \sum _{j=1}^{x} \left( X_{ij\lambda } - \mu _\lambda \right) ^2}, \end{aligned}$$

where $X_{ij\lambda }$ represents the pixel intensity at spatial coordinates (*i*, *j*) and spectral band $\lambda$, and $\mu _\lambda$ is the spatial mean of the spectral band $\lambda$:

3 $$\begin{aligned} \mu _\lambda = \frac{1}{yx} \sum _{i=1}^{y} \sum _{j=1}^{x} X_{ij\lambda }. \end{aligned}$$

The cumulative spatial standard deviation within each window is calculated as:

4 $$\begin{aligned} {S}_{W_k} = \sum _{\lambda = s}^{e-1} \sigma _\lambda , \end{aligned}$$

where $W_k$ denotes the *k*-th window of a fixed width *w*. The optimal spectral range $(s^*, e^*)$ is selected by maximizing the cumulative standard deviation score over all possible window positions:

5 $$\begin{aligned} (s^*, e^*) = \arg \max _{s, e} {S}_{W_k}. \end{aligned}$$

Three additional dimensionality reduction algorithms are implemented to evaluate the performance of the STD-based method, consisting of two widely used band selection methods, MI-based and Shannon entropy-based, and the RBS method.

#### Mutual information-based band selection

MI is a measure that quantifies the dependency between two random variables that are processed simultaneously. In HSI, MI is used to evaluate how much information a specific spectral band $\lambda _i$provides about a reference. A higher MI value indicates a stronger relationship between the spectral data and the reference, making the band more relevant for classification tasks^53^. The hypercube is first reshaped into a two-dimensional matrix $X' \in \mathbb {R}^{(y \cdot x) \times \lambda }$, where each row corresponds to a pixel and each column to a spectral band. The pixel-wise mean intensity across spectral bands is computed as:

6 $$\begin{aligned} \mu = \frac{1}{\lambda } \sum _{l=1}^{\lambda } X'[:, l], \end{aligned}$$

and discretized into a finite number of intensity bins. Similarly, each spectral band $\lambda$ is discretized independently using histogram binning. The mutual information between each spectral band $\lambda$ and the pixel-wise mean intensity $\mu$ is then estimated using:

7 $$\begin{aligned} I(\lambda ; \mu ) = \sum _{v \in \mathcal {V}_\lambda } \sum _{u \in \mathcal {V}_\mu } p(v, u) \log _2 \left( \frac{p(v, u)}{p(v)p(u)} \right) , \end{aligned}$$

where $\mathcal {V}_\lambda$ and $\mathcal {V}_\mu$ represent the discretized intensity levels in band $\lambda$ and in $\mu$, respectively. The variables $p(v, u)$, $p(v)$, and $p(u)$ denote the joint and marginal probability distributions. The cumulative mutual information within each spectral window is computed as:

8 $$\begin{aligned} {S}_{W_k} = \sum _{\lambda = s}^{e-1} I(\lambda ; \mu ), \end{aligned}$$

where $W_k$ denotes the $k$-th window of width $w$, starting at spectral index $s$, and $e = s + w$. The optimal spectral window is then selected using equation (5).

#### Shannon entropy-based band selection

Shannon entropy is a statistical measure of uncertainty within a probability distribution. It quantifies the variability of pixel intensities inside a spectral band. Spectral bands with high entropy are therefore characterized by increased information content and may indicate a unique or distinctive spectral feature^54^. For the Shannon entropy, the hypercube is reshaped in the same way as for MI into a two-dimensional matrix $X' \in \mathbb {R}^{(y \cdot x) \times \lambda }$. For each band $\lambda$, the reflectance values across all pixels are discretized into a histogram with $B$ bins. The Shannon entropy of the intensity distribution in band $\lambda$ is computed as:

9 $$\begin{aligned} H(\lambda ) = -\sum _{b=1}^{B} p_b \log _2(p_b), \end{aligned}$$

where $p_b$ denotes the normalized probability of intensity values falling into the $b$-th histogram bin. The cumulative entropy within each spectral window is calculated as:

10 $$\begin{aligned} {S}_{W_k} = \sum _{\lambda = s}^{e-1} H(\lambda ), \end{aligned}$$

where $W_k$ denotes the $k$-th window of width $w$, starting at spectral index $s$, and $e = s + w$. The optimal spectral window is then selected using equation (5).

#### Random band selection

To guarantee robust and repeatable results of the structured band selection methods, the RBS is used as a baseline to exclude the influence of chance. For each RBS run, a spectral window with a fixed width $w$ = 20 nm is selected uniformly at random along the spectral axis. To account for variability due to randomness, each method is executed ten times, and the classification performance and computation time are then reported as the mean and standard deviation across all runs. These metrics allow for evaluating not only performance and robustness but also computational efficiency.

#### Tissue dataset

**Fig. 3:**
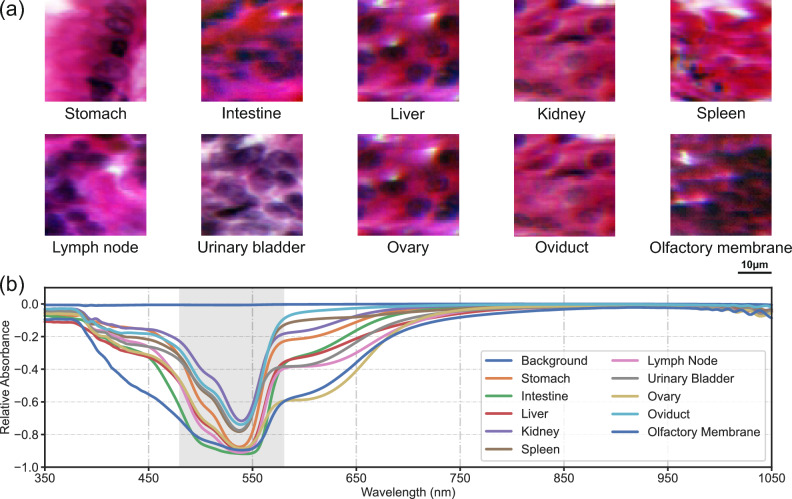
(**a**) Pseudo-RGB images of organ tissue samples stained with H&E and scanned using the HSI setup at 100$\times$ magnification, with spatial dimensions 64$\times$64. The scale bar represents 10 $\mu$m. (**b**) Median spectra of each organ type. The shaded gray region indicates the spectral range selected by the STD-based method, corresponding to the region of highest variance across the wavelength bands.

Ten tissue samples from human organs (stomach, intestine, liver, kidney, spleen, lymph node, urinary bladder, ovary, oviduct, and olfactory membrane) are selected, stained with hematoxylin and eosin (H&E), and mounted on microscopic slides for scanning as shown in Fig. 3(a). Hematoxylin stains the nuclei dark violet, while eosin stains the extracellular matrix and the cytoplasm pink. In addition, a background scan is included as a baseline to distinguish tissue-specific signals from potential noise and artifacts. These samples show high spectral similarity, as shown in Fig. 3(b), which increases the classification task complexity, demonstrating the effectiveness of using the STD for band selection. The gray region (470 nm - 570 nm) illustrates a predefined spectral range selected by the STD-based method, representing the area with the highest variation.

Each sample is scanned in the y-direction over a total distance of 32 $\mu$m with a step size of 0.5 $\mu$m, resulting in 64 scanned lines. This process generates every hypercube with spatial dimensions of 64$\times$64 (y scanning lines, x pixel) and $\times$1004 ($\lambda$) wavelength bands. The entire dataset is used in its raw state, and preprocessing and denoising were deliberately avoided to preserve subtle spectral features that may be relevant to biological interpretation. While denoising can reduce spectral noise, it may also suppress diagnostically useful absorption characteristics, especially in narrow bands^55–57^.

### Neural network and data preparation

**Fig. 4:**
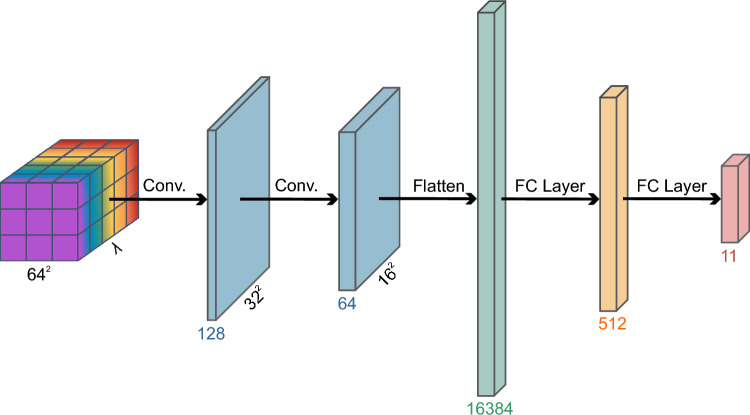
Neural network architecture used in this study for organ tissue classification. $\lambda$ represents the spectral channels. The models are trained with spectral widths of 20 nm, 50 nm, 100 nm, and 731 nm.

The neural network architecture consists of two convolutional layers and two fully connected (FC) layers, as shown in Fig. 4. Each convolutional layer features a 3$\times$3 kernel, starting with the hypercube as the input layer. After each convolutional layer, a non-linear activation function (ReLU) and a max pooling operation with a stride of 2, to reduce the spatial dimensions. The resulting feature maps are flattened into a one-dimensional vector before passing through two FC layers. The first FC layer, containing 512 nodes, applies another ReLU activation function to introduce non-linearity. The final FC layer produces the prediction, with the number of nodes equal to the number of human organ tissue samples and the background.

This well-established and straightforward architecture is designed to emphasize the STD-based method, ensuring effective classification while minimizing complexity. The CNN architecture is intentionally kept straightforward, influenced by the limited number of input spectral bands after dimensionality reduction and the relatively small dataset size. In biomedical imaging, where labeled data is typically limited and often requires expert annotations, deep networks are more prone to overfitting. Moreover, simpler architectures ensure faster training and better suitability for applications in real-time or resource-constrained environments. Based on these considerations, a minimal and interpretable architecture is selected.

**Fig. 5:**
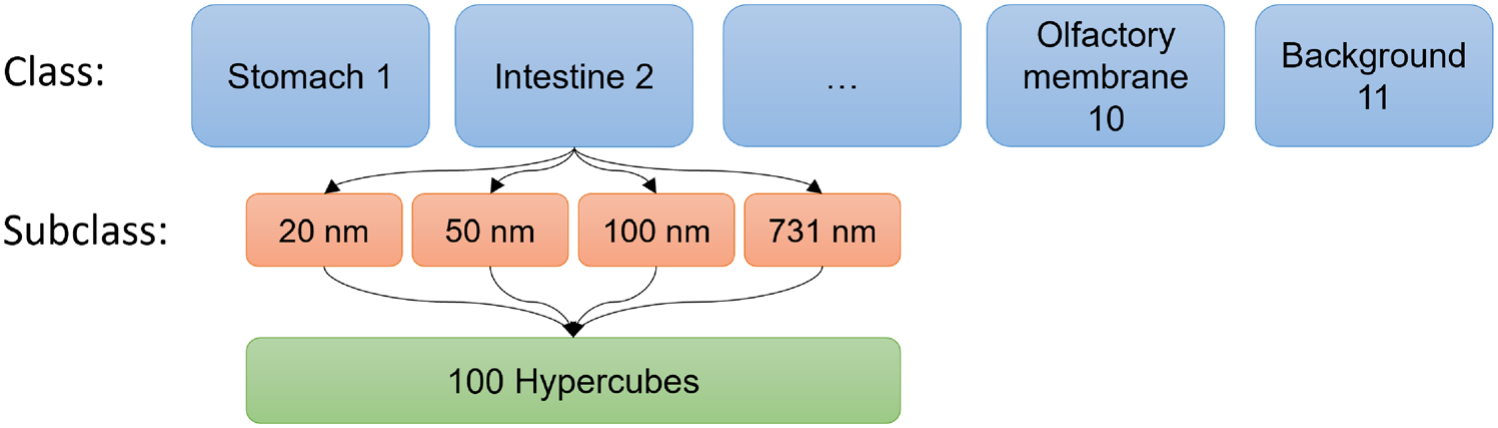
The training dataset for each organ class is prepared by selecting 100 hypercubes per predefined spectral band.

The dataset is categorized into 11 classes: stomach, intestine, liver, kidney, spleen, lymph node, urinary bladder, ovary, oviduct, olfactory membrane, and background, each serving as a label for classification. These classes are further divided into predefined spectral bands for later STD estimation: 20 nm, 50 nm, 100 nm, and the entire spectral range of 731 nm. Each subset within these spectral bands contains 100 hypercubes, as shown in Fig. 5. This means that for training on a specific spectral band, the dataset comprises 1100 hypercubes (11 classes $\times$ 100 hypercubes per spectral band subclass), which are randomly split into a training dataset (880 hypercubes) and a test dataset (220 hypercubes).

The neural network applies the cross-entropy loss function (CrossEntropyLoss), specifically designed for multi-class classification tasks. Training is carried out with the Adam optimizer and incorporates learning rate decay, starting at an initial rate of $10^{-4}$. A batch size of 2 is utilized, and early stopping is applied to halt training after 15 epochs if the validation loss fails to decrease, thus preventing overfitting. All training and testing are conducted on the same computer (NVIDIA GeForce RTX 3060).

## Results

**Fig. 6:**
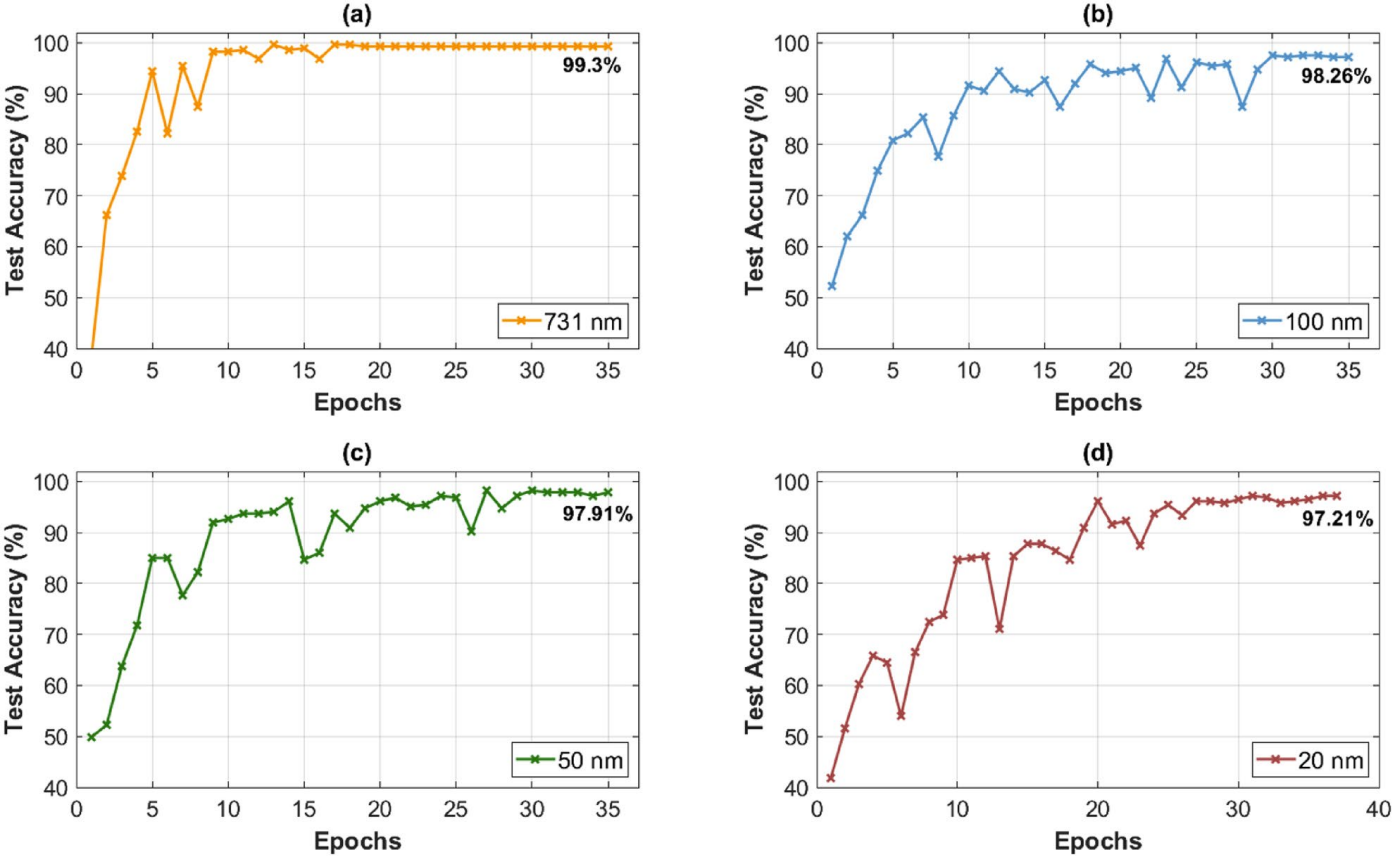
Classification accuracy across training epochs for organ tissue using different spectral bands of (**a**) 731 nm, (**b**) 100 nm, (**c**) 50 nm, and (**d**) 20 nm using the STD-based method.

The classification results demonstrate the efficiency of the STD-based method with predetermined spectral bands as a dimensionality reduction method. This approach reveals that most spectral information is redundant and that focusing on a few unique spectral features can significantly reduce computational load and processing time while maintaining high accuracy for the classification of human organ tissue. Fig. 6 shows representative runs for 731 nm, 100 nm, 50 nm, and 20 nm. The entire spectral range of 731 nm achieves a classification accuracy of 99.30% after 17 training epochs, as shown in Fig. 6(a).

Reducing the spectral range down to predefined bands of 100 nm, 50 nm, and 20 nm results in a slight reduction in classification accuracy, as shown in Fig. 6(b), (c), and (d). This suggests that even with a limited spectral band of unique features, the model can distinguish effectively between different tissue types despite their similar spectral characteristics. Also, with a narrow spectral band of 20 nm, the accuracy remains high at 97.21%, only decreasing by approximately 2% compared to the full range. Larger ranges of 50 and 100 nm reach classification accuracies of 97.91% and 98.26%, respectively, after approximately 35 training epochs. This indicates that the essential spectral features are sufficient for successful classification in HSI tasks, and high dimensionality is not essential.

The outcomes of the dimensionality reduction using the STD-based method provide meaningful benefits, as listed in Table 1. Defined spectral bands with distinctive features enable substantial data size reduction. Therefore, a spectral band of 100 nm leads to a reduction of 86.7% and up to 97.3% when using only 20 nm, all while achieving high classification accuracy. The reduced data size also contributes to faster computation times (epoch times) during training of the CNN. The computation times per epoch decrease to 211.25 s for 100 nm, 210.67 s for 50 nm, and 210.52 s for 20 nm compared to 235.86 s for 731 nm, which yields up to a 10.74% reduction in training time per epoch. It is important to note that reductions below 100 nm do not reduce training time significantly but do maximize the data reduction.

**Table 1:** Comparison of data size reduction, computation time per epoch, classification accuracy across spectral bands of 731 nm, 100 nm, 50 nm, and 20 nm using the STD-based method.

Spectral Band (nm)	Data Size Reduction (%)	Epoch Time (s)	Classification Accuracy (%)
731	-	235.86	99.30
100	86.7	211.25	98.26
50	93.9	210.67	97.91
20	97.3	210.52	97.21

Overall, selecting a spectral window of 20 nm for the band selection provides the best performance in the classification scenario by achieving an optimal balance between data size reduction and classification accuracy. Although the accuracy is slightly lower compared to 50 nm and 100 nm, the data size reduction is significantly higher by 10.6%, which results in a 97.3% reduction in total data size. This indicates no direct correlation between accuracy and data size reduction.

**Fig. 7:**
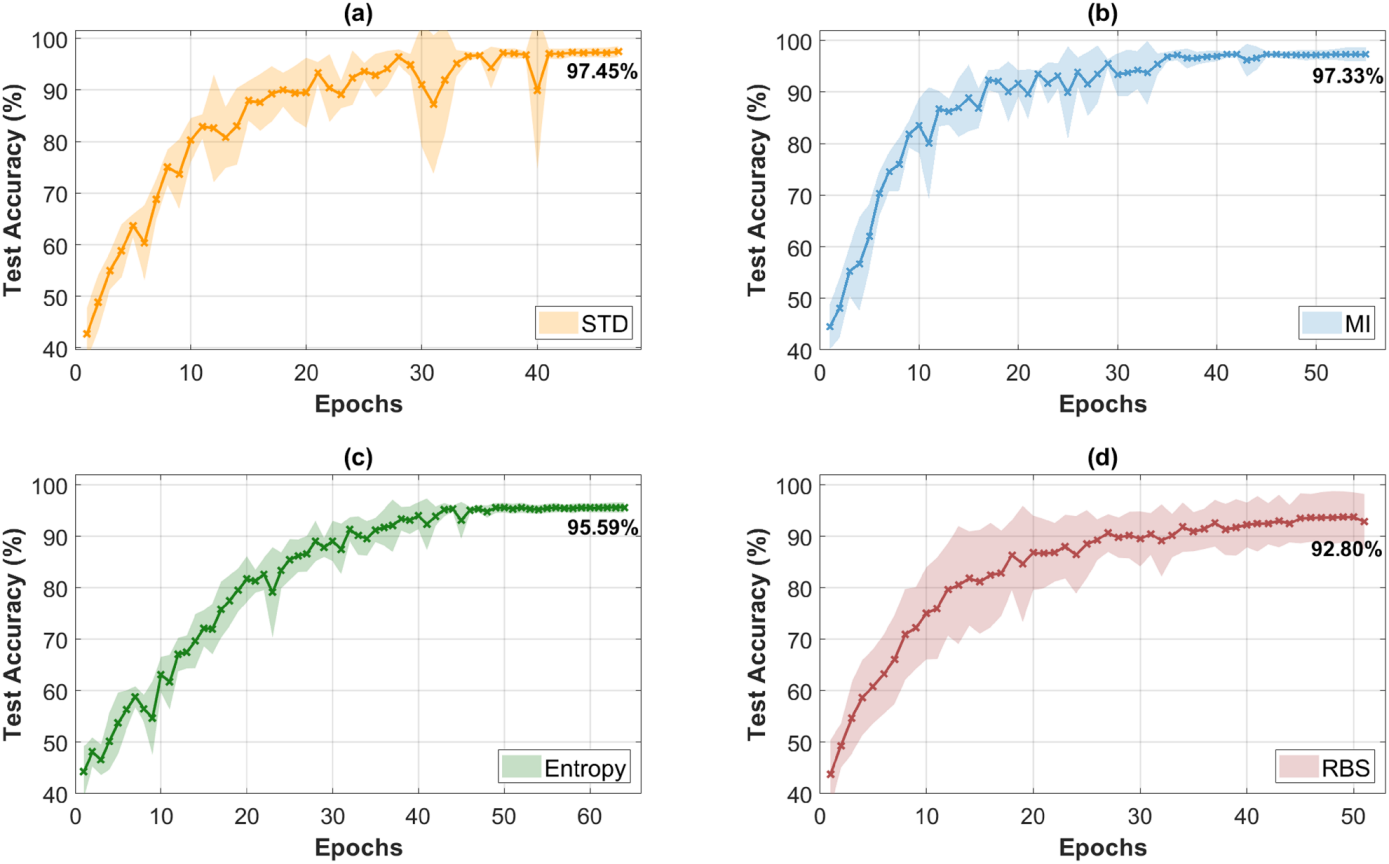
Average classification accuracy across training epochs of organ tissue classification using (**a**) the STD-based method, (**b**) MI, (**c**) Shannon entropy, and (**d**) RBS. The shaded envelopes represent the standard deviation across ten training runs.

To further validate the performance of the STD-based band selection, a comparative analysis is conducted using two established band selection methods: MI and Shannon entropy, as well as RBS, which serves as a control to verify the significance and repeatability of the results. Each method is evaluated across ten independent training runs, and the resulting classification accuracies are averaged per epoch to ensure fair comparison. The standard deviation (as the statistical measure) is visualized as an envelope for each method to represent the variance across runs, as shown in Fig. 7.

The STD-based method achieves a higher classification accuracy of 97.45%, when averaged over ten training runs, than its single run at 20 nm of 97.21%, as shown in Table 1. It also outperforms MI (97.33%), Shannon entropy (95.59%), and RBS (92.80%), while using fewer training epochs on average. This demonstrates the effectiveness of the STD-based method in both classification accuracy and training efficiency, reducing computational load significantly. Moreover, the standard deviation across training runs consistently decreases over time for all methods except RBS, indicating stable convergence and reliable model behavior when using systematic band selection approaches. In contrast, the RBS method exhibits high variance and lower training accuracy across all epochs, demonstrating the random nature of its spectral band selection. This highlights the importance of statistically grounded and data-driven methods for robust classification. The unstable performance of RBS further confirms that the results of the STD-based method, MI, and Shannon entropy are from meaningful feature selection rather than chance. Table 2 summarizes the average epoch time and classification accuracy for each band selection method.

**Table 2:** Comparison of average time per epoch and classification accuracy for different band selection methods: STD-based method, MI, Shannon entropy, RBS.

Method	Epoch Time ± std (s)	Classification Accuracy ± std (%)
STD	215.81 ± 0.74	97.45 ± 1.06
MI	214.58 ± 0.75	97.33 ± 1.32
Shannon Entr.	213.44 ± 0.41	95.59 ± 1.00
RBS	210.87 ± 1.57	92.80 ± 5.40

Despite having a higher average epoch time (215.81 s ± 0.74 s), compared to MI (214.58 s ± 0.75 s) and Shannon entropy (213.44 s ± 0.41 s), the training converges in fewer epochs, as shown in Fig. 7. This shows that the STD-based method is a viable alternative, offering high classification accuracy with comparable training time. The RBS does not specifically target spectral bands with distinct spectral features. As a result, it shows the lowest average epoch time (210.87 s ± 1.57 s), but at the cost of lower classification accuracy and higher variability. This comparison highlights the importance of a systematic and reliable band selection method for capturing the most relevant spectral information, ensuring consistent classification performance. It confirms that selecting spectral bands based on their variability and significance, as realized by the STD-based method, is an effective strategy for achieving high classification accuracy while reducing the computation time and data size. Spectral ranges of 50 and 100 nm are not tested for MI, Shannon entropy, and the RBS method, as 20 nm is identified as the optimal solution for high classification accuracy and significant data size reduction.

## Discussion

Handling high-dimensional data presents significant challenges in HSI^58^. Dimensionality reduction offers great potential for many applications where processing speed and accuracy are crucial. Research demonstrated that using only a few selected spectral bands with distinct features yields results comparable to the entire spectrum^59^. Band selection, in particular, is an effective way to reduce dimensionality while maintaining the original spectral characteristics. However, estimating the appropriate number of spectral bands is crucial to avoid losing essential information or including redundant information.

In this study, we presented a statistical band selection strategy based on the standard deviation, which was evaluated and compared to established methods such as MI, Shannon entropy, and RBS. The STD-based method demonstrated superior performance in both classification accuracy and reducing computational load, even when using narrow spectral windows. These findings highlight the robustness and consistency of the STD-based method as a dimensionality reduction method and confirm that most spectral information is redundant and that targeted band selection can greatly reduce the computational load and computation time while maintaining a high classification accuracy. Preprocessing and denoising of the hypercubes can be beneficial in reducing spectral noise and allow for spectral bands smaller than 20 nm with potentially similar accuracy. However, these steps are time-consuming and may alter characteristic features in the spectrum, potentially affecting results. Therefore, preprocessing and denoising were deliberately avoided to preserve these features and enable rapid data processing. In biomedical settings where fast, interpretable, and low-resource solutions are essential, the ability to achieve high classification accuracy using a simple, reproducible method is highly valuable. Our findings indicate that high classification performance can be achieved without resorting to complex models, and that traditional statistical measures, when applied strategically, can outperform deeper architectures under real-world constraints^60,61^.

As a result, applying the STD for spectral bands smaller than 20 nm was not feasible, as noise within the data was considered a distinct feature. For instance, the spectral band of 950 nm to 1050 nm was targeted as the characteristic feature, as shown in Fig. 3(b). While more complex neural architectures may further improve performance^62,63^, we emphasize that our STD-based band selection method can integrate into any model and be intentionally paired with a minimal network to demonstrate its general effectiveness. Future research could explore the use of combined dimensionality reduction techniques to further optimize classification tasks. Additionally, reinforcement learning methods could automatically determine the balance between optimal spectral band size, data size reduction, and classification performance^64^.

## Conclusion

This study presented an intuitive hypercube classification method using a straightforward neural network and a band selection based on the STD. HSI scans of human organ tissue were chosen to emphasize the method’s capability to differentiate between highly similar spectral characteristics, demonstrating its effectiveness in classification tasks despite significant dimensionality reduction. The STD-based method was applied to the spectral profiles of each hypercube with predefined spectral bands of 100 nm, 50 nm, and 20 nm, selecting areas with the most significant variability. Moreover, hypercubes with the complete spectral range (731 nm) were included to compare the classification performance. The reduced hypercubes were subsequently classified by organ type using a neural network. The results show that dimensionality reduction using band selection only has a minor influence on classification accuracy. Narrowing the spectral range to 100 nm decreases the data size by 86.7% while maintaining a high classification accuracy of 98.26% compared to 99.30% for the entire spectral range. Even narrower spectral bands of 50 nm or 20 nm achieve high classification accuracies with 97.91% and 97.21% (97.45% when averaged over ten runs) while significantly reducing the data size by 93.9% and 97.3% compared to the original size. Comparisons with established methods like MI and Shannon entropy confirm that the STD-based method is a robust and reliable dimensionality reduction method, outperforming both techniques in classification accuracy and computational efficiency. This makes it a suitable candidate for fast and reliable HSI processing, especially in biomedical applications where rapid analysis is essential. Our findings indicate that most of the spectral information is redundant and does not necessarily enhance the classification accuracy, and highlight the potential of simple, data-driven band selection strategies like the proposed STD-based method to enable fast, accurate, and resource-efficient hyperspectral image classification without sacrificing performance, even under significant dimensionality reduction.

## Data Availability

The datasets generated and/or analyzed during the current study are not publicly available due to institutional data-sharing policies at the TranslaTUM Center for Translational Cancer Research. However, they can be made available from the corresponding author, Wolfgang Kurz, upon reasonable request, in accordance with institutional regulations and data-sharing policies.
